# Admission Severity of Atrial-Fibrillation-Related Acute Ischemic Stroke in Patients under Anticoagulation Treatment: A Systematic Review and Meta-Analysis

**DOI:** 10.3390/jcm11123563

**Published:** 2022-06-20

**Authors:** Catarina Garcia, Marcelo Silva, Mariana Araújo, Mariana Henriques, Marta Margarido, Patrícia Vicente, Hipólito Nzwalo, Ana Macedo

**Affiliations:** 1 Faculty of Medicine and Biomedical Sciences, University of Algarve, 8005-139 Faro, Portugal; a63572@ualg.pt (C.G.); a63504@ualg.pt (M.S.); a63584@ualg.pt (M.A.); a63507@ualg.pt (M.H.); a63508@ualg.pt (M.M.); a63510@ualg.pt (P.V.); amamacedo@ualg.pt (A.M.); 2 Algarve Biomedical Center, 8005-139 Algarve, Portugal; 3 Stroke Unit, Algarve University Hospital Center, 8000-386 Algarve, Portugal

**Keywords:** ischemic stroke, atrial fibrillation, oral anticoagulation, stroke severity

## Abstract

Background: In non-valvular-associated atrial fibrillation (AF), direct oral anticoagulants (DOAC) are as effective as vitamin K antagonists (VKA) for the prevention of acute ischemic stroke (AIS). DOAC are associated with decreased risk and severity of intracranial hemorrhage. It is unknown if different pre-admission anticoagulants impact the prognosis of AF related AIS (AF-AIS). We sought to analyze the literature to assess the association between pre-admission anticoagulation (VKA or DOAC) and admission severity of AF-AIS. Methods: A Systematic literature search (PubMed and ScienceDirect) between January 2011 to April 2021 was undertaken to identify studies describing the outcome of AF-AIS. Results: A total of 128 articles were identified. Of 9493 patients, 1767 were on DOAC, 919 were on therapeutical VKA, 792 were on non-therapeutical VKA and 6015 were not anticoagulated. In comparison to patients without anticoagulation, patients with therapeutical VKA and under DOAC presented with less severe stroke (MD −1.69; 95% CI [−2.71, −0.66], *p* = 0.001 and MD −2.96; 95% Cl [−3.75, −2.18], *p* < 0.00001, respectively). Patients with non-therapeutical VKA presented with more severe stroke (MD 1.28; 95% Cl [0.45, 2.12], *p* = 0.003). Conclusions: In AF-AIS, patients under therapeutical VKA or DOAC have reduced stroke severity on admission in comparison to patients without any anticoagulation, with higher magnitude of protection for DOAC.

## 1. Introduction

Acute ischemic stroke (AIS) is a major cause of disability and death worldwide [1]. Atrial fibrillation (AF) is the most common cardioembolic etiology, associated with 15 to 30% of all cases of AIS [2,3]. Without treatment, AF is associated with a five-fold increased risk of AIS [4,5,6]. The burden of AF related AIS (AF-AIS) is well characterized. AF-AIS is associated with higher mortality, greater disability, prolonged hospitalization, and a lower chance of being discharged home [7,8,9,10,11,12,13,14,15]. Oral anticoagulants (OAC), namely vitamin K antagonists (VKA) and direct oral anticoagulants (DOAC), reduce the risk of AIS [4,7,16,17,18]. In non-valvular AF, the efficacy of DOAC parallels that of other OAC. Moreover, in comparison to patients taking VKA, patients under treatment with DOAC have reduced frequency and severity of intracerebral hemorrhage, the most lethal complication of anticoagulation therapy [19].

Reflecting the presence of residual thromboembolic risk, AIS can occur even in patients under adequate OAC. Nevertheless, the annual incidence of AF-AIS in patients without OAC (43.4 per 1000 person-years) is much higher than the 9.4 and 10.4 per 1000 person-years from patients under DOAC and warfarin, respectively [20].

It is unknown if different pre-admission OAC can have different impacts on AF-AIS prognosis. We sought to review the literature to assess the association between pre-admission anticoagulation (VKA or DOAC) therapy and initial severity in patients with AF-AIS.

## 2. Materials and Methods

This protocol follows the Preferred Reporting Items for Systematic Reviews and Meta-Analysis Protocols (PRISMA-P) guidelines [21,22]. The review was registered within the International Prospective Register of Systematic Reviews on 17 April 2021 (PROSPERO ID: CRD42021249531).

### 2.1. Literature Research

A systematic literature review was performed using PubMed and ScienceDirect databases. The search strategy in this study was as follows: (“atrial fibrillation”) AND (“prior anticoagulation” OR “prestroke anticoagulation” OR “anticoagulation before stroke” OR “preceding anticoagulation”) AND (“stroke severity” OR “stroke outcome”).

### 2.2. Data Extraction

Two investigators independently screened articles (title, abstract, full articles) to assess eligibility. Titles, names of authors, year of publication, study design, sample and control group size, study characteristics, outcomes and results were extracted and recorded in a Microsoft Excel spreadsheet. Therapeutical and non-therapeutical dosages of the drugs involved were defined based on the criteria of each author in the respective articles. All duplicated articles were removed. Any disagreement was resolved by a third reviewer. 

### 2.3. Study Selection and Quality Assessment

To assess the impact of preadmission anticoagulation on stroke severity, analysis was limited to studies specifically designed or aimed to compare AF-AIS outcomes between patients taking or not taking any specific OAC. To reduce the risk of information or selection bias as well as socioeconomic status as a confounding bias, studies not specifically designed to compare the outcome with a control group were excluded.

We also excluded articles written in a language other than English. The methodological quality of each study was assessed in a qualitative way. Risk of bias was assessed according to the Cochrane Handbook for Systematic Reviews of Interventions guidelines [23].

### 2.4. Statistical Analysis

The Review Manager (RevMan) software (version 5.4, Cochrane Collaboration) was used for data analysis. For the meta-analysis, the included outcomes were measured using the same metrics in a minimum of three studies. Stroke severity score on admission, measured by National Institutes of Health Stroke Scale (NIHSS), was analyzed in subgroups (VKA therapeutical, VKA non-therapeutical and DOAC). For continuous variables, we used Hozo et al.’s [24,25] formula to estimate mean and variance values from the median, interquartile range and size of the samples. Results were reported with effect sizes and 95% confidence intervals (CI). For continuous variables, this was determined using the inverse variance random-effects model. For dichotomous variables, CI and odds ratio (OR) were measured using the Mantel–Haenszel random-effects model. The OR and mean differences were represented on a forest plot. Heterogeneity across studies was quantified based on Higgins statistics, considering I^2^ ≥ 50% evidence of a substantial heterogeneity in our meta-analysis. Studies not reporting AIS stroke severity on admission with NIHSS as a mean or a median were excluded from the meta-analysis. In addition, a random-effects approach was selected to extrapolate results as a small number of studies—not functionally identical (performed by researchers operating independently)—were included. This model considers the existence of differences between the studies that go beyond simple sample variability [26]. We intended to assess publication bias through funnel plot asymmetry [23].

## 3. Results

### 3.1. Study Selection

**Figure 1:**
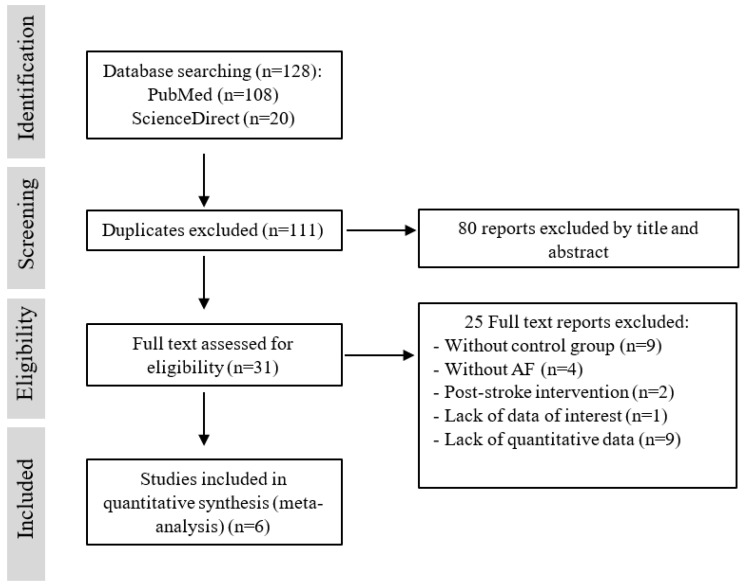
PRISMA-P flowchart of the study inclusion process. PRISMA indicates Preferred Reporting Items for Systematic Reviews and Meta-Analyses.

PRISMA flowchart diagram (Figure 1) outlines the selection and inclusion process. The initial search yielded 128 studies. The number of studies after deduplication was 111, and 31 fulfilled criteria for full-text reading. From those, 25 were excluded. Reasons for exclusion were the absence of a control group (*n* = 9), prior anticoagulation due to conditions other than AF (*n* = 4), post-stroke intervention (*n* = 2), absence of data regarding AIS outcome (*n* = 1) and lack of quantitative data reporting stroke severity (NIHSS) as a mean or a median (*n* = 9).

### 3.2. Study Characteristics

Six eligible studies reporting on stroke severity, enrolling a total of 9493 participants, 3478 anticoagulated patients and 6015 controls, were included. Regarding anticoagulated patients, 1767 were on DOAC and 1711 on VKA. From the latter, only 919 were therapeutically anticoagulated, considering data provided by the authors. Baseline characteristics of the participants involved are summarized in Table 1.

### 3.3. Quality Assessment

The quality assessment of each study is described in Table A1 and Appendix A. We considered that five studies had a low risk of bias and one had a moderate risk. None of the included articles had a high risk of bias. According to the Cochrane Handbook for Systematic Reviews of Interventions, the funnel plot asymmetry can only be used when there is a minimum of 10 studies included in the meta-analysis, otherwise the test has a low power to distinguish chance from real asymmetry [23]. As we have only included 6 studies in our meta-analysis, publication bias was not assessed.

### 3.4. General Sociodemographic and Risk Factors

As seen in Table 2, the median age (range 71–80) as well the distribution of gender, vascular risk factors and the thrombotic risk (CHADS2 score 2–3) was in general comparable between the studies and between treatment arms (DOAC versus therapeutic VKA versus non-therapeutic VKA versus controls).

### 3.5. Stroke Severity

**Figure 2:**
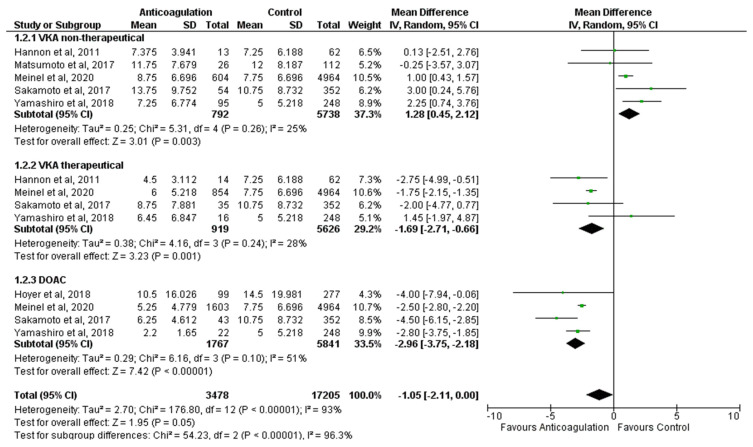
Stroke severity. Forest plot showing the effects of anticoagulation versus control (no anticoagulation) on stroke severity, measured by NIHSS.

Six studies were included in the quantitative analysis (Figure 2). Of these, five studies reported results on VKA therapy [13,27,28,29,30] and four studies reported results on DOAC therapy [12,13,27,28]. In five studies, there was a difference of AIS severity between therapeutical VKA and non-therapeutical VKA [13,27,28,30]. None of the studies presented a comparison of admission severity between patients taking DOAC and VKA.

### 3.6. VKA Non-Therapeutical

Five studies reported on stroke severity in patients with non-therapeutical VKA. The studies by Meinel et al. [13] (MD 1.00; 95% CI [0.43, 1.57]), Sakamoto et al. [27] (MD 3.00; 95% CI [0.24, 5.76]) and Yamashiro et al. [28] (MD 2.25; 95% CI [0.74, 3.76]) showed that the severity was lower in the group of patients without any OAC compared to patients with non-therapeutical VKA. Hannon et al. [29] and Matsumoto et al. [30] did not find any significant statistical differences between the two groups, with MD 0.13; 95% CI [−2.51, 2.76] and MD −0.25; 95% CI [−3.57, 3.07], respectively.

Overall, compared with control groups (87.87%, *n* = 5738), patients with non-therapeutical VKA on admission (12.13%, *n* = 792) had significantly higher NIHSS scores (MD 1.28; 95% Cl [0.45, 2.12], *p* = 0.003). I^2^ was 25%, showing a low heterogeneity in this dataset.

### 3.7. VKA Therapeutical

Therapeutical INR levels were provided in four studies. Hannon et al. [29] and Meinel et al. [13] revealed differences between patients with therapeutical VKA and those without OAC, MD −2.75; 95% CI [−4.99, −0.51] and MD −1.75; 95% CI [−2.15, −1.35], respectively. The other two studies, by Sakamoto et al. [27] and Yamashiro et al. [28], did not show a significant difference between the two groups, MD −2.00; 95% CI [−4.77, 0.77] and MD 1.45; 95% CI [−1.97, 4.87], respectively.

Overall, in AF-AIS, patients with therapeutical VKA (14.04%, *n* = 919) had significantly lower NIHSS scores (MD −1.69; 95% CI [−2.71; −0.66], *p* = 0.001) compared to control groups (89.96%, *n* = 5626), suggesting a protective effect associated with therapeutical VKA. I^2^ was 28%, showing a low heterogeneity in this dataset.

### 3.8. DOAC

Four studies reported results about anticoagulation therapy with DOAC. In all studies, DOAC therapy (23.23%, *n* = 1767) was associated with lower NIHSS scores (MD −2.96; 95% Cl [−3.75, −2.18], *p* < 0.00001) compared to control groups (76.77%, *n* = 5841) [12,13,27,28]. I^2^ was 51%, showing a substantial heterogeneity in this dataset.

The meta-analysis taking into account all subgroups of OAC (VKA non-therapeutical, VKA therapeutical and DOAC) did not show any significant statistical difference between the anticoagulated and control groups (MD −1.05; 95% CI [−2.11, 0.00], *p* = 0.05). However, I^2^ was 93%, showing a substantial heterogeneity in this dataset.

## 4. Discussion

In this study we assessed associations between pre-admission use of OAC and severity of AF-AIS. The main finding of our analysis was that, despite differences in the mechanism of action, prior use of OAC is associated with lower severity of AF-AIS on admission. Clot formation inhibition depends on the international normalized ratio (INR) for VKA and on plasma concentration for DOAC [31]. It is known that patients on VKA [32] or DOAC [33] therapy present with smaller infarct volumes on diffusion-weighted magnetic resonance imaging. There is some evidence showing an increased susceptibility to fibrinolysis in the presence of VKA or DOAC [31,32]. Surprisingly, patients with non-therapeutic INR on admission present with higher AIS severity. One might speculate that suboptimal INR control may reflect a higher burden of medical comorbidities, poor medication compliance (other than anticoagulation), worse health literacy or even later hospital presentation. All these factors may adversely influence stroke outcome [34]. Another factor that might help to explain these results is VKA’s mechanism of action: these drugs inhibit the production of several factors dependent on vitamin K, including protein C and protein S [35]. In subtherapeutic INR, there is an insufficient suppression of coagulation such that it cannot overcome the reduction in protein C anticoagulant activity [27]. As a result, when compared with no treatment, the use of subtherapeutic VKA may result in larger thrombi formation [27]. Although no direct comparison between DOAC use and VKA use was presented, the magnitude of the protection was higher for patients taking DOAC in comparison to patients taking VKA (MD −2.96; 95% Cl [−3.75, −2.18], *p* < 0.00001 versus MD −1.69; 95% CI [−2.71; −0.66], *p* = 0.001). Real life meta-analysis to compare VKA to DOAC impact on AF-AIS outcomes would be interesting as cost-effectiveness is an important factor for decision.

There are some limitations that are worthwhile to discuss. Population heterogeneity is a reality as we analyzed observational studies using different patient selection. For instance, the samples were not controlled for pre-hospital delay which can determine the extent of severity at the time of admission to the hospital. There is a discrepancy between the number of controls and anticoagulated patients, and the number of therapeutically anticoagulated patients is considerably small. Standardized blood plasma or serum concentration therapeutic ranges for DOAC have not been established and quantitative test results have not been correlated with clinical outcomes [36]. Assumption of anticoagulation status for the DOAC group was based on interview or medical prescription as blood drug concentration has not been measured. Nevertheless, adequate adherence to DOAC therapy has been shown to have a beneficial effect on stroke severity on admission [28] and concurrent pro-thrombotic risk factors appear to be relevant when AIS occurs despite adequate OAC [37].

Only six studies were suitable for meta-analysis; publication bias was not assessed (funnel plot asymmetry has low power if *n* < 10) [23]. This means that we cannot exclude that such bias might have affected our results.

## 5. Conclusions

In conclusion, our systematic review demonstrated that pre-stroke anticoagulation is associated with less severe presentation of AF-AIS with apparent higher protection in patients under DOAC.

## Figures and Tables

**Table 1:** Description of the studies included in the meta-analysis.

Study	Year	Type of Study	Inclusion Criteria	Exposure				Outcomes	Risk of Bias
				Without OAC	DOAC	Therapeutical VKA	Non-Therapeutical VKA		
Meinel et al. [13]	2020	Observational retrospective	AIS patients with AF (AF diagnosed either before or after stroke onset) aged ≥18 years	*n* = 4964	*n* = 1603	INR > 1.7 *n* = 854	INR < 1.7 *n* = 604	NIHSS at admission	Low
Sakamoto et al. [27]	2017	Observational retrospective	Patients with AIS or TIA with known AF	*n* = 352	*n* = 43	*n* = 35	*n* = 54	NIHSS at admission	Moderate
Yamashiro et al. [28]	2018	Observational retrospective	Patients with AF who developed AIS or TIA	*n* = 248	*n* = 22	INR ≥ 2 *n* = 16	INR < 2 *n* = 95	NIHSS at admission	Low
Hoyer et al. [12]	2018	Observational retrospective	Patients with newly detected AF or known AF admitted for AIS	*n* = 277	*n* = 99	-		NIHSS at admission	Low
Hannon et al. [29]	2011	Observational prospective	Patients with new stroke events and AF (known or new)	*n* = 62	-	INR 2–3 *n* = 14	INR < 2 or > 3 *n* = 13	NIHSS < 72 h	Low
Matsumoto et al. [30]	2017	Observational retrospective	AF patients who suffered AIS	*n* = 112	-	-	*n* = 26	NIHSS at admission	Low

VKA: Vitamin K Antagonists; AIS: Acute Ischemic Stroke; AF: Atrial Fibrillation; DOAC: Direct Oral Anticoagulants; NIHSS: National Institute of Health Stroke Scale; OAC: Oral Anticoagulants; TIA: Transient Ischemic Stroke; IS: Ischemic Stroke.

**Table 2:** Population and vascular risk factors characteristics in studies included in the meta-analysis.

	DOAC				VKA Therapeutical				VKA Non-Therapeutical					Control Group					
	Meinel et al. [13]	Sakamoto et al. [27]	Yamashiro et al. [28]	Hoyer et al. [12]	Meinel et al. [13]	Sakamoto et al. [27]	Yamashiro et al. [28]	Hannon et al. [29]	Meinel et al. [13]	Sakamoto et al. [27]	Yamashiro et al. [28]	Matsumoto et al. [30]	Hannon et al. [29]	Meinel et al. [13]	Sakamoto et al. [27]	Yamashiro et al. [28]	Matsumoto et al. [30]	Hoyer et al. [12]	Hannon et al. [29]
**Mean age, y**	79.8	-	-	79.2	80.7	-	-	-	82.1	-	-	76	-	79.4	-	-	76	79.4	-
**Median age, y**	-	78 (71–82)	71 (63–81)	-	-	80 (74-88)	80 (73–84)	77 (70–80)	-	79 (75–86)	80 (72–85)	-	76 (69–80)	-	78 (70–85)	74 (67–82)	-	-	77 (66–83)
**Sex,** **(% female)**	46%	49%	40.6%	52.5%	44%	54%	31.3%	64.3%	54%	41%	39%	45%	46%	49%	42%	36.7%	49%	54.5%	50%
**Hypertension** **(%)**	87%	74%	77.3%	86.9%	87%	69%	87.5%	71.4%	90%	67%	67.4%	86%	38.5%	82%	61%	64.9%	73%	85.6%	53%
**Diabetes mellitus** **(%)**	25%	26%	45.5%	28.3%	28%	17%	37.5%	21.4%	27%	20%	35.8%	24%	23.1%	21%	16%	20.2%	27%	31.8%	3.3%
**Hyperlipidemia** **(%)**	66%	33%	45.5%	30.3%	67%	54%	43.8%	71.4%	64%	29%	39%	41%	46.2%	60%	29%	36.7%	35%	27.1%	29.5%
**Smokers** **(%)**	12%	5%	10.5%	1%	10%	6%	28.6%	42.9%	8%	15%	25.6%	22%	61.5%	13%	17%	24.3%	30%	51%	57.9%
**CHADS2 score**	-	3 (2–4)	2 (1–3.3)	-	-	3 (2–4)	3 (2.3–4)	-	-	3 (2–4)	2 (2–3)	3.1 ± 1.5	-	-	2 (1–3)	2 (1–2)	2.8 ± 1.4	-	-

CG: control group; DOAC: Direct Oral Anticoagulants; tVKA: therapeutical Vitamin K Antagonists; ntVKA: non-therapeutical Vitamin K Antagonists.

## Data Availability

Not applicable.

## Appendix A

**Table A1:** Risk of bias Assessment.

	A	B	C	D	Risk of Bias		
Meinel et al., 2020 [13]					Low	**A**	Confounding bias
Sakamoto et al., 2017 [27]					Moderate	**B**	Selection bias
Yamashiro et al., 2018 [28]					Low	**C**	Information bias
Hoyer et al., 2018 [12]					Low	**D**	Reporting bias
Hannon et al., 2011 [29]					Low		
Matsumoto et al., 2017 [30]					Low		Low
							Some concerns
							High
